# How Epiphytic Are Epiphytes? Field Evidence for Flexible Substrate Use in Tropical Cloud Forest Plants

**DOI:** 10.1002/ece3.74310

**Published:** 2026-09-07

**Authors:** Maria Judith Carmona‐Higuita, Jan Philipp Gerber, Helena Bertelmann, Ana María Benavides, Edith Rosario Clemente Arenas, Jhon Fernando Taborda, Laura Méndez, Glenda Mendieta Leiva, Alexander Zizka

**Affiliations:** ^1^ Department of Biology University of Marburg Marburg Germany; ^2^ Marburg Center for Biodiversity Monitoring and Modelling University of Marburg Marburg Germany; ^3^ Jardín Botánico de Medellín Medellín Colombia; ^4^ Faculty of Biological Sciences National University of San Marcos Lima Peru; ^5^ Biodiversity Research Institute (IMIB) University of Oviedo‐CSIC‐Principality of Asturias Mieres Spain; ^6^ Department of Organismal and Systems Biology University of Oviedo Oviedo Asturias Spain

**Keywords:** growth habit, intraspecific variation, tropical montane forest, understory, vascular plants

## Abstract

Substrate use in vascular plants is commonly treated as a categorical trait where a species is either epiphytic or terrestrial. Yet many species grow on both substrates, and the degree of epiphytism varies continuously within and among species. We quantified substrate use in 27 species traditionally classified as epiphytic across two tropical montane cloud forests in Colombia and Peru, recording 483 instances of substrate use, and compared field‐derived Epiphytic Values (EV) with literature‐derived estimates. In the field, 17 species grew exclusively epiphytically, while 10 also grew terrestrially, with EVs ranging from 43% to 97%. Literature‐derived EVs consistently overestimated substrate fidelity relative to field observations, particularly single‐source classifications, with mean absolute differences of up to 32%. Our results challenge treating ‘epiphytic/terrestrial’ as a categorical trait and support a continuous ‘Epiphytic Value’ per species.

## Graphical abstract

In the field, 63% of the species grew exclusively epiphytically, while 37% also grew terrestrially. Literature‐derived epiphytic values consistently overestimated substrate fidelity relative to field observations, particularly single‐source classifications, with mean absolute differences of up to 32%. Our results challenge treating ‘epiphytic/terrestrial’ as a categorical trait and support a continuous ‘Epiphytic Value’ per species.

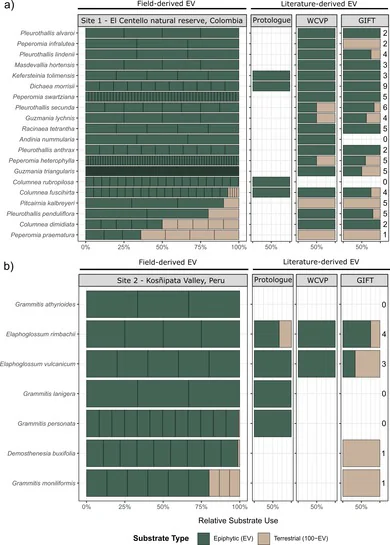

## 1 Introduction

Vascular epiphytes contribute about 10% to the global vascular plant species richness (Zotz et al. 2021) and up to 50% in some tropical cloud forests (Kelly et al. 2004). Due to the varying light and moisture conditions in the forest canopy, specific adaptations are associated with epiphytic growth; for instance, velamen radicum (sponge‐like root tissue), atmospheric water uptake, CAM photosynthesis and a specialised root microbiome (Zotz 2016). While these adaptations are beneficial for living in the canopy, they are costly and may therefore reduce the competitiveness of epiphytes with terrestrial plants for growing on the ground (Hoeber and Zotz 2022).

Despite the clear definition of epiphytes as ‘plants growing non‐parasitically on other plants for the entire life without contact to the ground’ (Zotz 2016), implementing this concept in the field is ambiguous, because many species grow epiphytically and terrestrially. This is possible because organic material can accumulate on branches and provide soil‐like growing conditions, enabling ‘terrestrial’ species to grow as accidental epiphytes, but also because some species are able to grow in both substrate types. For instance, in filmy ferns (Hymenophyllaceae; Zotz and Einzmann 2023), and in orchids and ferns of the Peruvian páramo (Acuña‐Tarazona et al. 2022), most species have a substrate preference, but strict specificity is rare.

Various attempts have tried to capture the variation in substrate use through categorical classifications, for instance as ‘true epiphytes’, ‘hemiepiphytes’, ‘facultative epiphytes’ and ‘accidental epiphytes’ (Benzing 2008). While these classifications have proven useful, a more flexible framework reflecting the biological variability within species is necessary to reconstruct the evolution of epiphytism and to assess species' vulnerability to Global Change (Carmona‐Higuita et al. 2024). Therefore, the ‘Epiphytic Value (EV)’ has recently been proposed, as the proportion in which a species grows epiphytically in comparison to lithophytically (on bare rock) or terrestrially (Zotz et al. 2023). This approach has the potential to overcome the limitations of categorical systems, but has so far been mostly used to synthesise literature descriptions (Zotz et al. 2023; Zotz and Einzmann 2023) leaving the true range of EV in the field undocumented and likely underestimated.

Here we present field data from two tropical montane cloud forests to (a) document the variability in EV for 27 species traditionally labelled ‘epiphytic’ and (b) compare their field‐derived EVs with their literature‐derived EVs.

## 2 Methods

**FIGURE 1:**
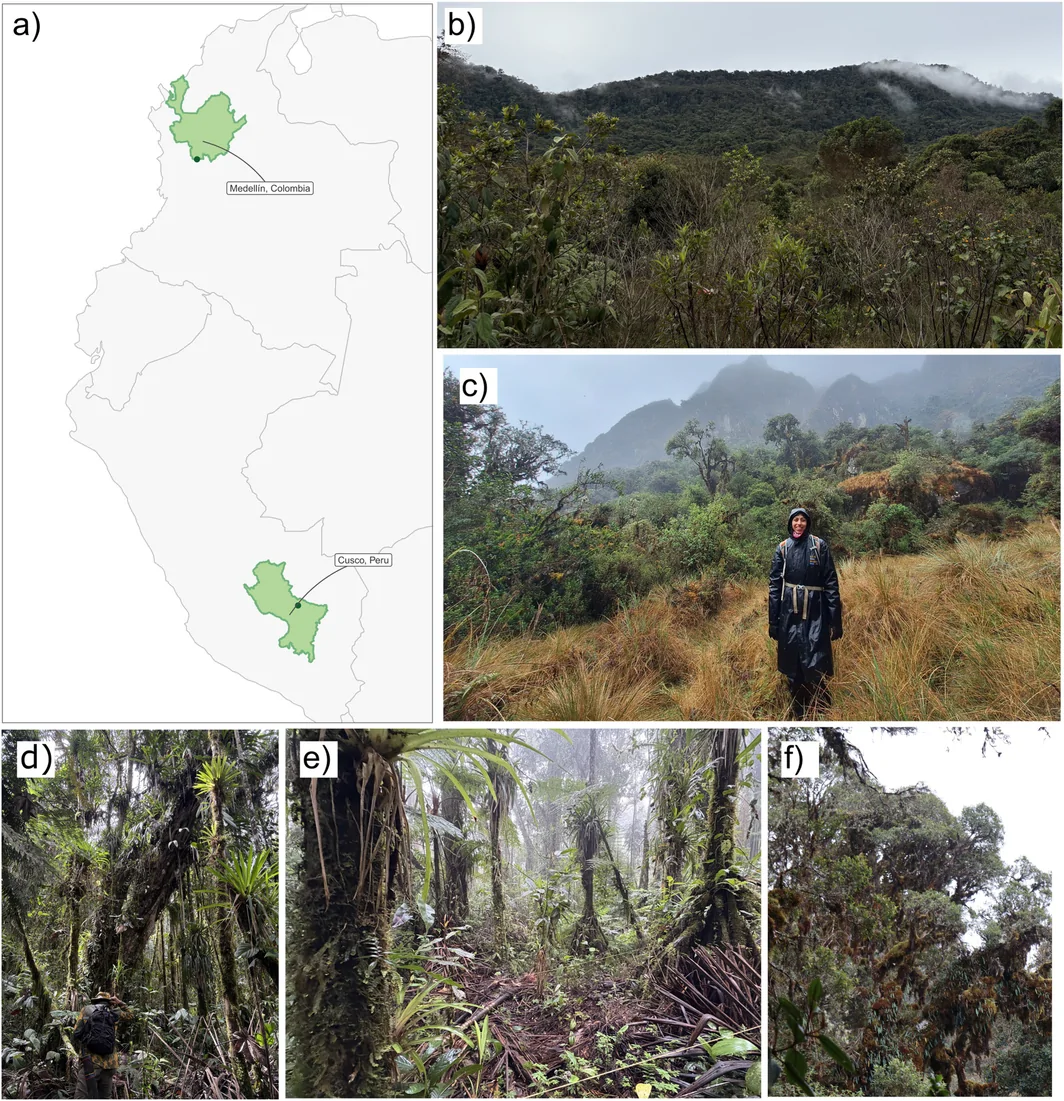
Overview of the forest structure at the sampling sites. (a) Location of the sampling sites in Colombia (site 1) and Peru (site 2). (b) Typical vegetation at ‘El Centello’ natural reserve, Jardin, Colombia, located on the western slope of the Andes. (c) Typical vegetation at the upper elevation limit of ‘Trocha Unión’ forest, Kosñipata Valley, Cusco, Peru, located on the eastern slope of the Andes in the highlands of Manu National Park. (d, e) Examples of study species growing on different substrates at site 1. (f) Examples of study species growing on trees at site 2.

We sampled two sites of tropical cloud forests rich in epiphytes (Figure 1): (a) Site 1 in ‘El Centello’ natural reserve in Colombia (75.85° W, 5.50° N), in May and June 2025. Site 1 has a bimodal seasonality that peaks in April and November and lies between 2400 m and 2700 m a.s.l., where the typical tree stand has a multi‐layered canopy up to 30 m in height, but is dominated by a high density of small‐to‐medium sized trees (Carmona‐Higuita and Benavides 2026); and (b) Site 2 in Manu National Park in the Kosñipata Valley in Peru (71.63° W, 13.10° S), in April 2025. Site 2 has a rainy season between November and March and lies at the tree line between upper tropical montane cloud forest and puna at 3622 m a.s.l., and the trees of the close‐canopy forest reach up to 12 m in height.

At both sites, we randomly placed understory plots across the landscapes (5 × 5 m and 2 m height; Krömer et al. 2007), restricting sampling to the understory where substrate use variation between epiphytic and terrestrial is most likely to occur, as species growing in the upper canopy are expected to be mainly epiphytic regardless of local conditions. Plots were located with a minimum distance of 40 m between them, ensuring that each plot contained trees and terrestrial substrate and no visible signs of human disturbance. We covered 25 plots at site 1 and 5 plots at site 2. The number of plots was lower at site 2 due to the steep terrain and the limited size of the forest (located in the tree line). In all plots, we recorded the substrate use (epiphytically/on trees vs. terrestrially/on soil; we did not include lithophytic individuals growing on bare rock in the analyses, because they only occurred at site 2 and cannot clearly be assigned as epiphytic or terrestrial) documented as epiphytes in an authoritative reference (Zotz et al. 2021). At site 2 we included all vascular plant species, whereas at site 1, due to the higher diversity, we recorded only species in the families Orchidaceae, Piperaceae, Bromeliaceae and Gesneriaceae. We limited the sampling to plants that we could identify to species level based on vegetative and reproductive characters when present and ignored all seedlings. At site 1 where the number of individuals was low, we counted individuals and at site 2, where the number of individuals was high and often in clonal stands, we recorded the presence of each species per substrate within each plot rather than counting individuals, given the challenge of separating clonal stands visually. Coverage estimates were noted in the field to characterise abundance but were not used in the EV calculation.

We then calculated the EV for each species with three or more records as the percentage of cases in which the species was using each substrate. To compare our field measurements with literature‐derived EV, we obtained descriptions of substrate use from three types of literature sources, representing different levels of information aggregation: (a) the species' protologues, as a single literature source (Table S1); (b) the World Checklist of Vascular Plants (WCVP; Govaerts et al. 2021), which provides a single, expert‐based life form classification (Table S2), and (c) the Global Inventory of Floras and Traits (GIFT; Weigelt et al. 2020), which provides life form descriptions from multiple literature references per species (Table S2). We then followed the methodology suggested by Zotz and Einzmann (2023) to convert categorical literature references into continuous EVs. In short, this meant an EV of 100% for species exclusively described as epiphyte, 50% for species described as ‘epiphyte and/or terrestrial’, and 0% for species exclusively described as terrestrial, with intermediate values for mixed descriptions (see Zotz and Einzmann 2023 for details). For the GIFT data, we first converted the description from each available literature reference into an EV value following Zotz and Einzmann (2023) and then calculated the mean EV for each species.

## 3 Results

**FIGURE 2:**
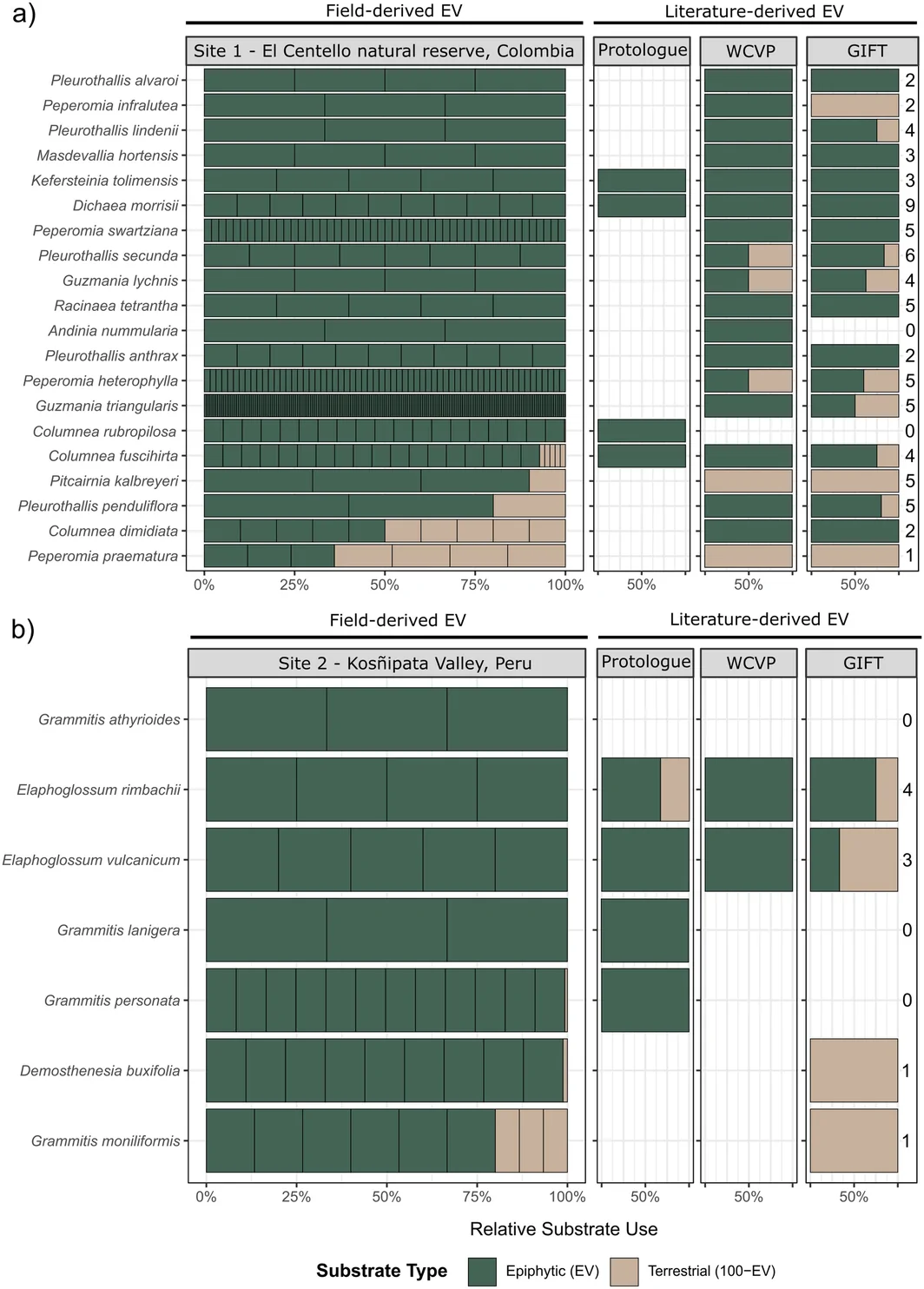
Field‐derived and literature‐derived epiphytic values (EVs; percentage of records in which a species grew epiphytically) for 27 species at two montane cloud forest sites. (a) Site 1: ‘El Centello’ natural reserve, Jardín, Colombia. (b) Site 2: Kosñipata Valley, Cusco, Peru. The left‐hand panels show field‐derived EVs based on 483 substrate use records and the right‐hand side panels show literature‐derived EVs from three sources (protologues, WCVP and GIFT). Each horizontal bar represents one species found in the field, where black lines separate individuals for site 1 and coverage estimates for site 2. For the GIFT database, the numbers to the right indicate the amount of literature references per species found in the database. Missing bars indicate that no substrate use information EV was available for that species and source. Species are ordered by decreasing field‐derived EV within each site, where EV% indicates the percentage of records in which a species was found growing epiphytically and the remaining proportion (100−EV%) represents terrestrial records.

In the field, we recorded a total of 483 instances of substrate use (individuals and cover estimates; Table S3). The overall average of EV was high at 90% (Figure 2). However, substrate use varied, with 17 species (63%) growing exclusively epiphytically (EV = 100%) and 10 species (37%) growing epiphytically and terrestrially (Figure 2). For these 10 species, the EV ranged from 43% to 97%, with a mean of 75%. The proportion of species with mixed substrate use was similar between sites (7 out of 20 species at site 1 and 3 out of 7 species at site 2; Figure 2).

In the literature, we found life form information convertible to EV for 8 species from their protologues, for 21 species from WCVP and for 22 species from GIFT (Table S3). For GIFT, the number of literature references per species ranged from 0 to 9 (median 4). The proportion of species with an EV of 100% was 87.5% in the protologues, 76.1% in WCVP and 36.4% in GIFT (Figure 2).

Field‐ and literature‐measurements agreed to classify species with an EV of 100% (exclusive epiphytes) for 5 species in case of the protologues, 12 species in the case of WCVP and 7 species in the case of GIFT. The mean absolute difference between field‐ and literature‐derived EVs was 8.4% for the protologues, 17.9% for WCVP and 31.8% for GIFT.

## 4 Discussion

Our results confirm previous literature‐derived EV assessment showing that most species have clear substrate use preferences but that many species can grow epiphytically and terrestrially (Figure 2; Zotz et al. 2023; Zotz and Einzmann 2023). The comparison between field‐ and literature‐derived EVs showed that (a) literature‐derived EVs based on single sources (the protologues and WCVP) underestimate the within‐species variability of substrate use; (b) literature‐derived EVs compiled from multiple references per species (GIFT) can better capture within species variability; and (c) even for multi‐reference databases, the overlap between field and literature‐derived EVs was generally poor. While (a) and (b) are of methodological relevance, (c) may reflect the intention of literature‐derived life form descriptions to capture general species properties, rather than local substrate use. We note that continuous EVs and traditional categorical classifications are not mutually exclusive: categories may reflect the general ecological tendency of a species, and morphological adaptations associated with epiphytic growth (e.g., velamen radicum; Zotz 2016) may correlate with higher substrate fidelity. Field‐derived EVs, by contrast, capture observed substrate use at a particular site, which may vary with local conditions, elevation, or sampling approach (Benzing 2008). Incidentally, two species of *Elaphoglossum* Schott ex J.Sm. occurring at site 2 in Peru, were included in a recent literature‐based classification of EV (Zotz et al. 2023) and our field‐EVs were 100% for both species, compared to 81% for *Elaphoglossum rimbachii* (Sodiro) Christ and 83% for *E. vulcanicum* Christ in that study. Furthermore, our field‐derived EV for *Grammitis moniliformis* (Lag.) Proctor from our site 2 in Peru was similar to the EV found in another field study at high elevations above 3200 m a.s.l. (Acuña‐Tarazona et al. 2022; 67% vs. 50%). Alternatively, the mismatch of field‐ and literature‐derived EVs may also indicate a different treatment of lithophytes (if they are included as ‘terrestrials’, EV decreases) or regional variation in life form possibly linked to elevation or moisture availability as has been suggested for *Aechmea distichantha* Lem. (Barberis et al. 2021).

Our results reveal some challenges with consistently sampling EVs in the field. In particular, modifications in the sampling protocol necessary to accommodate local field conditions can limit comparability among sites and species. Because of the high number and/or hard to separate clonal individuals (from asexual reproduction) at site 2, we recorded coverage per substrate rather than individual substrate use as at site 1. To improve the comparability of field‐derived EVs across sites and species in the future, we recommend using a standardised sampling unit. Ideally, this should be the ‘stand’ rather than coverage (Sanford 1968), as the sampling unit across studies, following explicit protocols for clonal species where individual delimitation is ambiguous (i.e., using the clonal colony or ‘genet’ as unit). We encourage researchers to report raw counts of individuals/stands per substrate alongside EVs to document sample size as this will allow post hoc reclassification and meta‐analyses as classification conventions evolve. Furthermore, future studies estimating field‐derived EVs can facilitate reproducibility and comparability by: (i) sampling across multiple sites and environmental conditions, as EVs may vary within and across species with elevation, moisture and forest structure; (ii) applying an explicitly defined minimum tree size criterion across sites to ensure that the available amount of epiphytic substrate is comparable; (iii) using the number of clonal stands (genet) as the sampling unit rather than coverage estimates per area, in cases where clonal growth makes individual delimitation ambiguous, because genet number can be standardised more reliably; and (iv) using a standardised sampling design in which all individuals encountered within the surveyed plots or sampling units are recorded, regardless of how many are found. Whether a minimum threshold of individuals/stands would improve EV reliability remains an open question; we tentatively suggest aiming for at least ten records per species distributed across independent sampling units, but the appropriate threshold likely depends on the species' substrate flexibility and the spatial scale of the study.

In summary, we take our results to emphasise that substrate use is more flexible than the broad categories such as ‘epiphyte’ and ‘terrestrial’ suggest. Locally measured EVs consistently differ from literature‐derived estimates, with single‐source classifications showing the greatest overestimation of substrate fidelity. This underscores the value of field‐based EV assessment as a complement to literature synthesis and highlights the need for standardised sampling protocols that will allow EVs to be compared across sites, taxa and environmental gradients.

## Author Contributions

**Maria Judith Carmona‐Higuita:** conceptualization (equal), data curation (equal), methodology (equal), supervision (supporting), writing – original draft (equal), writing – review and editing (supporting). **Jan Philipp Gerber:** data curation (equal), investigation (lead), methodology (equal), validation (equal), writing – review and editing (supporting). **Helena Bertelmann:** data curation (equal), funding acquisition (supporting), investigation (lead), methodology (lead), validation (equal), writing – review and editing (supporting). **Ana María Benavides:** funding acquisition (supporting), investigation (supporting), supervision (supporting), writing – original draft (equal), writing – review and editing (equal). **Edith Rosario Clemente Arenas:** data curation (equal), investigation (supporting), methodology (supporting), writing – review and editing (supporting). **Jhon Fernando Taborda:** data curation (supporting), investigation (supporting). **Laura Méndez:** data curation (supporting), software (supporting), writing – review and editing (supporting). **Glenda Mendieta Leiva:** conceptualization (equal), data curation (equal), funding acquisition (lead), project administration (lead), supervision (lead), validation (equal), writing – original draft (equal), writing – review and editing (equal). **Alexander Zizka:** formal analysis (lead), funding acquisition (lead), software (equal), visualization (lead), writing – original draft (lead), writing – review and editing (lead).

## Funding

M.J.C.‐H. acknowledges funding by the Deutscher Akademischer Austauschdienst (DAAD) (91893488). Fieldwork was additionally supported by a PROMOS (DAAD) stipend to H.B. and G.M.L. acknowledges funding by the German Research Foundation (CONTINENT, ME 5087/2‐1).

## Conflicts of Interest

The authors declare no conflicts of interest.

## Supporting information


**Table S1:** Protologue citations for the 20 study species for which original species descriptions were accessible, including full bibliographic reference and digital source. For 7 species there a not available protologue.

**Table S2:** Literature‐derived Epiphytic Values (EVs) for the 27 study species from multiple sources: taxonomic details from the World Checklist of Vascular Plants (WCVP; columns wcvp_*), life form classifications from protologues (protologue_life_form) and WCVP (wcvp_life_form), EV estimates from protologues (protologue_EV), WCVP (wcvp_EV), and GIFT (gift_EV), the number of literature references underlying each GIFT estimate (gift_nref) and the field‐derived EV (field_EV).

**Table S3:** Raw field substrate use records for the 27 study species at the two study sites (Site 1: El Centello natural reserve, Colombia; Site 2: Kosñipata Valley, Peru). Each row is one observation record; columns indicate species name, substrate type (Tree or Soil), genus, site, and whether the identification reached species level or remained as morphospecies (type). Records from Colombia represent individual plant counts; records from Peru represent species presence per plot.

## Data Availability

The data supporting this study is provided in the Tables [Link], [Link], [Link]. The analysis scripts have been archived in Zenodo and will be made publicly available upon publication.

## References

[ece374310-bib-0001] Acuña‐Tarazona, M. , K. Mehltreter , T. Toledo‐Aceves , V. J. Sosa , A. Flores‐Palacios , and M. Kessler . 2022. “Effects of Microenvironmental Factors on the Diversity and Composition of Fern and Orchid Assemblages in an Andean Paramo in Peru.” Flora 293: 152107. 10.1016/j.flora.2022.152107.

[ece374310-bib-0002] Barberis, I. M. , V. Y. Mogni , L. J. Oakley , C. Vogt , and D. E. Prado . 2021. “Biogeography of Different Life Forms of the Southernmost Neotropical Tank Bromeliad.” Journal of Biogeography 48, no. 8: 2085–2097. 10.1111/jbi.14137.

[ece374310-bib-0003] Benzing, D. H. 2008. Vascular Epiphytes: General Biology and Related Biota. Cambridge University Press.

[ece374310-bib-0004] Carmona‐Higuita, M. J. , and A. M. Benavides . 2026. “The Role of Host Tree Characteristics in Epiphyte Community in Northern Andean Cloud Forests: A Case Study in El Centello Nature Reserve.” Caldasia 48: e115388. 10.15446/caldasia.v48.115388.

[ece374310-bib-0005] Carmona‐Higuita, M. J. , G. Mendieta‐Leiva , J. A. Gómez‐Díaz , et al. 2024. “Conservation Status of Vascular Epiphytes in the Neotropics.” Biodiversity and Conservation 33, no. 1: 51–71. 10.1007/s10531-023-02730-8.

[ece374310-bib-0006] Govaerts, R. , E. Nic Lughadha , N. Black , R. Turner , and A. Paton . 2021. “The World Checklist of Vascular Plants, a Continuously Updated Resource for Exploring Global Plant Diversity.” Scientific Data 8, no. 1: 215. 10.1038/s41597-021-00997-6. 34389730 PMC8363670

[ece374310-bib-0007] Hoeber, V. , and G. Zotz . 2022. “Accidental Epiphytes: Ecological Insights and Evolutionary Implications.” Ecological Monographs 92, no. 4: e1527. 10.1002/ecm.1527.

[ece374310-bib-0008] Kelly, D. L. , G. O'Donovan , J. Feehan , S. Murphy , S. O. Drangeid , and L. Marcano‐Berti . 2004. “The Epiphyte Communities of a Montane Rain Forest in the Andes of Venezuela: Patterns in the Distribution of the Flora.” Journal of Tropical Ecology 20, no. 6: 643–666. 10.1017/S0266467404001464.

[ece374310-bib-0009] Krömer, T. , M. Kessler , and S. R. Gradstein . 2007. “Vertical Stratification of Vascular Epiphytes in Submontane and Montane Forest of the Bolivian Andes: The Importance of the Understory.” Plant Ecology 189, no. 2: 261–278. 10.1007/s11258-006-9182-8.

[ece374310-bib-0010] Sanford, W. W. 1968. “Distribution of Epiphytic Orchids in Semi‐Deciduous Tropical Forest in Southern Nigeria.” Journal of Ecology 56, no. 3: 697–705. 10.2307/2258101.

[ece374310-bib-0011] Weigelt, P. , C. König , and H. Kreft . 2020. “GIFT – A Global Inventory of Floras and Traits for Macroecology and Biogeography.” Journal of Biogeography 47, no. 1: 16–43. 10.1111/jbi.13623.

[ece374310-bib-0012] Zotz, G. 2016. Plants on Plants – The Biology of Vascular Epiphytes (Bd. 15). Springer Nature Link. 10.1007/978-3-319-39237-0.

[ece374310-bib-0013] Zotz, G. , L. Armenia , and H. J. R. Einzmann . 2023. “A New Approach to an Old Problem: How to Categorize the Habit of Ferns and Lycophytes.” Annals of Botany 132, no. 3: 513–522. 10.1093/aob/mcad128. 37642212 PMC10666995

[ece374310-bib-0014] Zotz, G. , and H. J. R. Einzmann . 2023. “How Epiphytic Are Filmy Ferns? A Semi‐Quantitative Approach.” Diversity 15, no. 2: 270. 10.3390/d15020270.

[ece374310-bib-0015] Zotz, G. , P. Weigelt , M. Kessler , H. Kreft , and A. Taylor . 2021. “EpiList 1.0: A Global Checklist of Vascular Epiphytes.” Ecology 102, no. 6: e03326. 10.1002/ecy.3326. 33713353

